# The effects of pressure on the energy landscape of proteins

**DOI:** 10.1038/s41598-018-20417-x

**Published:** 2018-02-01

**Authors:** Fabio Librizzi, Rita Carrotta, Judith Peters, Antonio Cupane

**Affiliations:** 1 Istituto di Biofisica, Consiglio Nazionale delle Ricerche, Via Ugo La Malfa 153, 90146 Palermo, Italy; 2 Université Grenoble Alpes, CNRS, LiPhy, 38000 Grenoble, France; 3 Institut Laue Langevin, 71 Avenue des Martyrs, CS 20156, F-38042 Grenoble, France; 4 Dipartimento di Fisica e Chimica, Università di Palermo, Viale delle Scienze, Ed. 18, 90128 Palermo, Italy

## Abstract

Protein dynamics is characterized by fluctuations among different conformational substates, i.e. the different minima of their energy landscape. At temperatures above ~200 K, these fluctuations lead to a steep increase in the thermal dependence of all dynamical properties, phenomenon known as Protein Dynamical Transition. In spite of the intense studies, little is known about the effects of pressure on these processes, investigated mostly near room temperature. We studied by neutron scattering the dynamics of myoglobin in a wide temperature and pressure range. Our results show that high pressure reduces protein motions, but does not affect the onset temperature for the Protein Dynamical Transition, indicating that the energy differences and barriers among conformational substates do not change with pressure. Instead, high pressure values strongly reduce the average structural differences between the accessible conformational substates, thus increasing the roughness of the free energy landscape of the system.

## Introduction

The peculiar dynamical properties of proteins arise from their complex nature, which can be well described in terms of a multi-minima energy landscape. In fact, proteins exist in a very large number of slightly different, hierarchically arranged, conformational substates (CS)^1,2^. At low temperature, each protein molecule is confined in one local minimum of the energy landscape, and the system behaves as a harmonic solid. On the contrary, at high temperature, typically above ~200 K, proteins start to fluctuate among different CS and this brings about a steep increase in all their dynamical properties, in particular in the Mean Square Displacements (MSD) of their atoms^3,4^. This phenomenon is usually referred to as Protein Dynamical Transition (PDT)^4^. The physical origin of the PDT and its relationship with the dynamical properties of hydration water and/or the external matrix, has raised debates in the literature, still being an open and intriguing question^2,4–16^. The motions arising above the PDT, which are specific for proteins, are believed to be important for functionality, since they give to protein molecules the flexibility which is necessary to perform their functions^1^.

PDT has been extensively studied during the last decades. Nevertheless, very little is known about the effects on it of pressure, the other important thermodynamic variable together with temperature. Studies of protein structural dynamics in general, and of the PDT in particular, as a function of pressure would be highly desirable^17^, both to help clarifying its physical origin and from a more biological point of view, since many biological systems live at high hydrostatic pressure^18,19^. Very few studies have addressed the problem of the pressure dependence of protein dynamics, and most of them have been concerned with samples in solution investigated near room temperature. In general, high pressure values lead to a reduction of internal protein dynamics^20–24^. To compensate this reduction, a higher protein flexibility at high pressure has been suggested as an adaptive strategy for organisms living in such conditions, like *Thermococcus Thioreducens*^25^ or *Thermococcus Barophilus*^26^. As a matter of fact, however, experimental studies of the pressure effects on the PDT and on the protein energy landscape are lacking, probably because of the relevant experimental challenges. In 2007 Meinhold *et al*.^27^ investigated, by molecular dynamics simulations, the dynamical properties and the PDT of a lysozyme-water system at different pressure values. In this study, the onset temperature for the PDT (*T*_*on*_) was found to not depend on pressure, which, on the contrary, had a strong reduction effect on the picosecond MSD of protein atoms, both below the PDT (in the “harmonic” temperature range) and at high temperature. Moreover, at high temperature, collective motions were found damped at high pressure, suggesting an increased roughness in the free energy landscape of the protein^27^. It must be noted, in this respect, that also in studies on the pressure dependence of protein folding processes, an increased roughness in the free energy landscape of proteins was suggested as a possible origin for the observed slowing down of the folding and unfolding kinetics at high pressure values^28–30^.

We report here on Elastic Incoherent Neutron Scattering (EINS) experiments on an ultraviscous mixture of Myoglobin (Mb), glycerol and water^31^, in a wide range of temperature (20–300 K) and pressure (20–5000 bar), aimed at the investigation of the pressure dependence of the dynamical properties of the protein. The choice of the sample (50% Mb, 33% D8-Glycerol, 17% D_2_O, see Materials for a detailed description of sample preparation) was motivated by the fact that we had to go to cryogenic temperature to investigate the PDT and the dynamical properties of the protein, while keeping the protein in a (very viscous) liquid-like environment, suitable for pressure transmission. The amount of water present in the sample was sufficient to allow the flexibility necessary for protein function^32^ and its high viscosity assures that overall protein diffusion and rotation have no influence on the dynamical properties measured in the time window of our experiment (~100 ps, see Materials and Methods). In this respect, it is worth of noting that the results that we obtained at low pressure are in excellent agreement with those relative to standard hydrated protein samples and in particular to myoglobin, as investigated by Doster *et al*.^4^ (see below).

As well known, EINS gives information on the MSD and on the dynamical properties of all non-exchangeable protein hydrogen atoms^33,34^. Since hydrogen atoms make up about half the atoms in a protein and are almost uniformly distributed within the molecule, this technique gives a global view of protein motions^35^. Only very recently, a dynamical transition, not present for hydrogen atoms, has been observed for the backbone atoms in an extremely dry per-deuterated protein sample^16^. On the contrary, for hydrated proteins, as it is in our sample, when the dynamical properties of non-exchangeable hydrogen atoms are compared to those of backbone atoms^36^ or of all atoms of the molecules^35,37^, analogous results are generally obtained. This holds, also, concerning our specific study, in investigations about the effects of pressure on protein dynamics^27^.

## Results and Discussion

**Figure 1:**
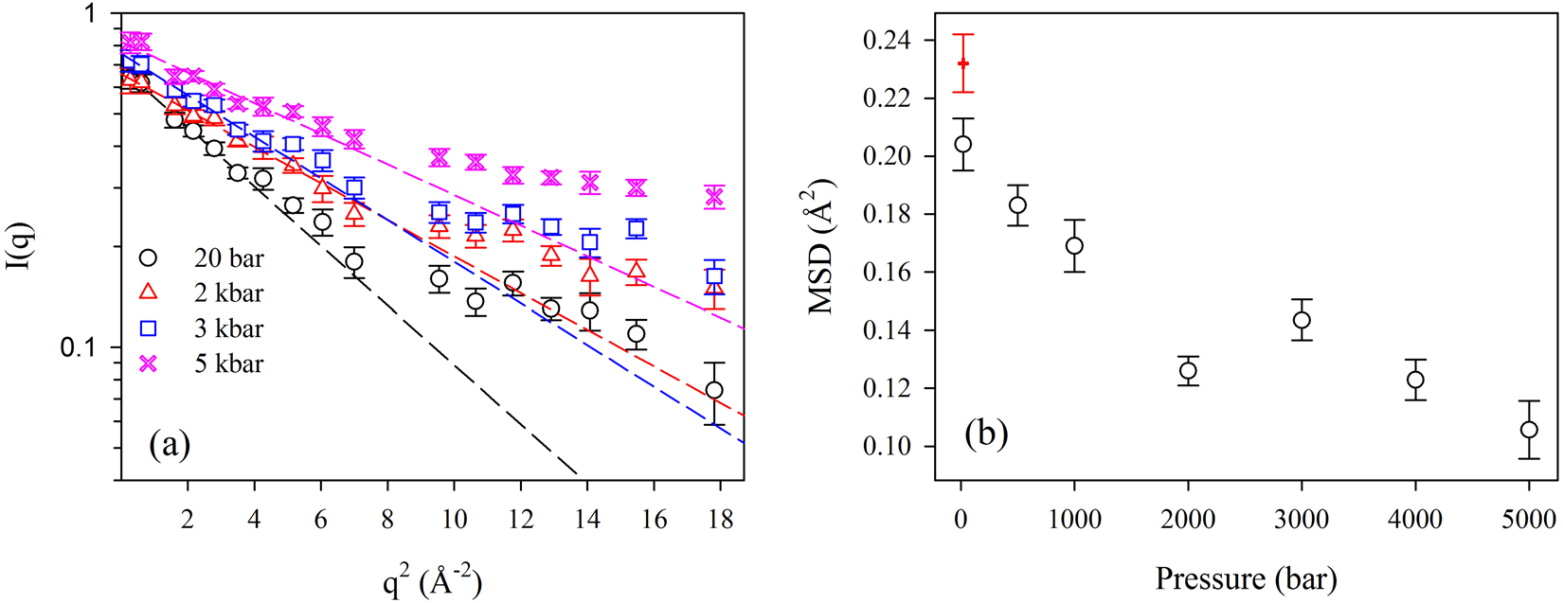
(**a**) Neutron scattering curves at the temperature of 295 K, for different pressure values; (**b**) MSD at 295 K, obtained within the framework of the Gaussian Approximation (i.e. from the slope of the straight lines shown in **a**).

Figure 1a reports the scattering curves obtained at 295 K, at different pressure values. The total MSD at the temperature of 295 K, obtained in the framework of the Gaussian Approximation^33^ (see Materials) in the q range 0–2 Å^−1^, are reported as a function of pressure in Fig. 1b. The values obtained at the lowest pressure are in good agreement with data on hydrated myoglobin powders^4^ in the whole temperature range (see Supplementary Information), confirming the appropriateness of our experimental conditions for the investigation of protein internal motions. As a general trend, in agreement with Le Châtelier’s principle^38^, when increasing the pressure we observe a sizeable reduction of molecular motions, except when going from 2 kbar to 3 kbar, where the MSD increases. This effect can be attributed to the onset of a pressure-induced denaturation, or partial unfolding of the protein. Both these findings, general reduction of protein mobility and pressure induced unfolding, are in agreement with previous studies^20–23,26^. The reduction of motions at high pressure can be related to an increase of the density in the hydration shell of proteins, as measured by Small Angle X-Ray Scattering^20^. Concerning the pressure-induced cold unfolding, it is a very well-known phenomenon, discovered more than a century ago^39^. It can be driven by a decrease in the occupied volume of protein molecules upon denaturation, due also to the presence of cavities in the folded form^40^, as in the case of myoglobin. In fact, a very complete study by Roche *et al*. clearly demonstrated the role of cavities for pressure driven protein unfolding^41^. Pressure induced unfolding has been already associated to an increase in the MSD, measured by EINS^22^, and the same effect is known for temperature denaturation^42^. As described in the Materials, measurements were performed on the same sample, from low to high pressure. Always on the same sample, at the end of the experiments, we measured again the elastic scattering at low pressure and high temperature. For this second measurement, the total MSD resulted significantly larger than at the beginning (the red point in Fig. 1b), thus confirming the pressure-induced denaturation.

**Figure 2:**
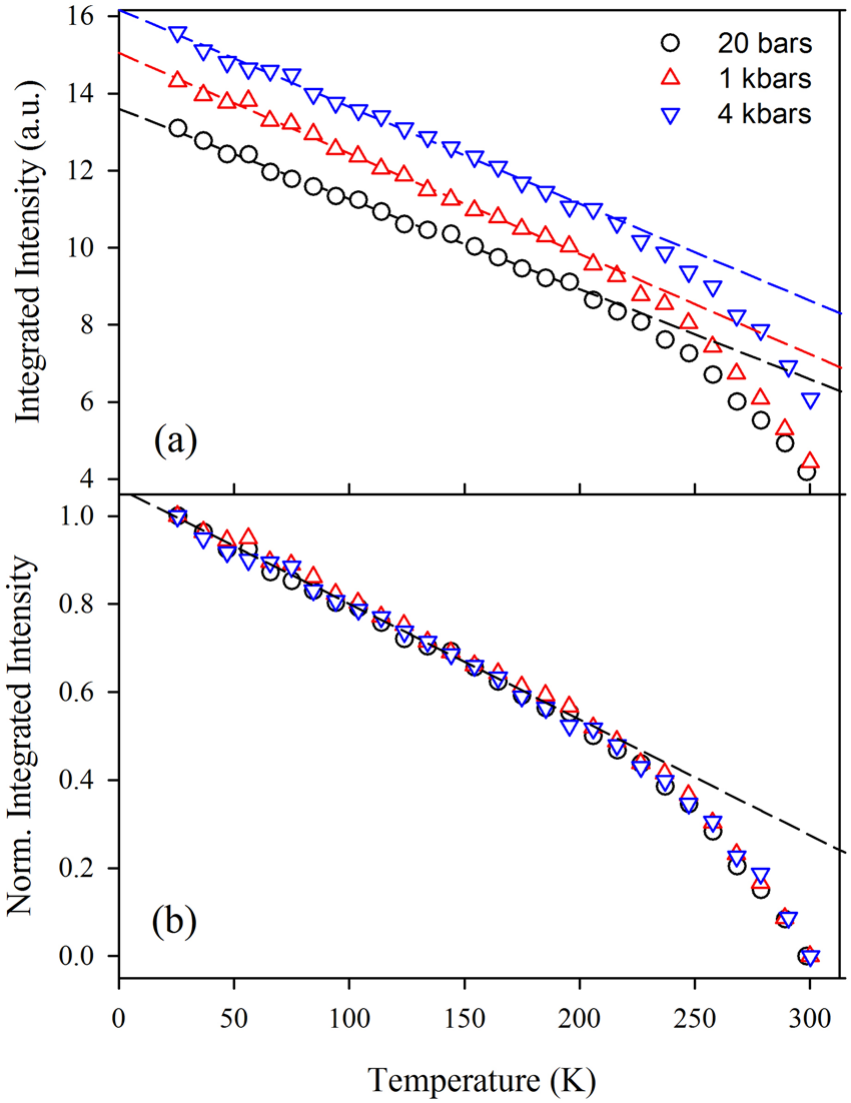
(**a**) Total scattered intensity as a function of temperature, for different pressure values. In (**b**), the same data are reported after normalization for their minimum and maximum value, according to eq. 1.

One of the purposes of our experiments was the investigation of the overall temperature dependence of the dynamical properties of the protein at the various pressure values. An important point, in particular, is the study of the pressure dependence of *T*_*on*_. For a first analysis, not conditioned by any model, we consider the total integrated scattered intensity, which is plotted in Fig. 2a as a function of temperature, for different pressures. As evident, the overall temperature dependence does not seem to be affected by pressure, as better shown in Fig. 2b, where the same data have been normalized for their maximum and minimum value, according to the following expression:

1 $${I}_{N}(T)=[I(T)-I(300K)]/[I(20K)-I(300K)]$$

All curves collapse on a unique pressure-independent master curve. This strongly suggests that *T*_*on*_ does not depend on pressure, in agreement with the above discussed molecular dynamics study^27^.

More detailed information can be obtained by analysing the data in the framework of the well-known two-state model^4^, i.e. assuming that each hydrogen atom can stay in two different wells, separated by a distance *d* and with a free energy difference Δ*G*. In fact, in spite of its simplicity, this model captures some important thermodynamic properties of proteins, since the two states can be considered as an average representation of their complex, multi-minima, free energy landscape. The scattering intensity as a function of *q* is given by^4^:

2 $$I(q)={e}^{-{\langle {\rm{\Delta }}{x}^{2}\rangle }_{0}{q}^{2}}\{1-2{p}_{1}{p}_{2}(1-\frac{\sin (qd)}{qd})\}$$

where $\langle {\rm{\Delta }}{{x}^{2}\rangle }_{0}$ is the MSD of the hydrogen atoms inside each single well and *p*_1_ and *p*_2_ are the populations of the two wells. The total MSD:

3 $${\langle {\rm{\Delta }}{x}^{2}\rangle }_{tot}=-{(\frac{d\mathrm{ln}\{I(q)\}}{d({q}^{2})})}_{q=0}={\langle {\rm{\Delta }}{x}^{2}\rangle }_{0}+\frac{{p}_{1}{p}_{2}{d}^{2}}{3}$$

is given by the sum of the MSD inside each single well ${\langle {\rm{\Delta }}{x}^{2}\rangle }_{0}$ and a second term, taking into account the existence of the hydrogen atoms in the two states.

**Figure 3:**
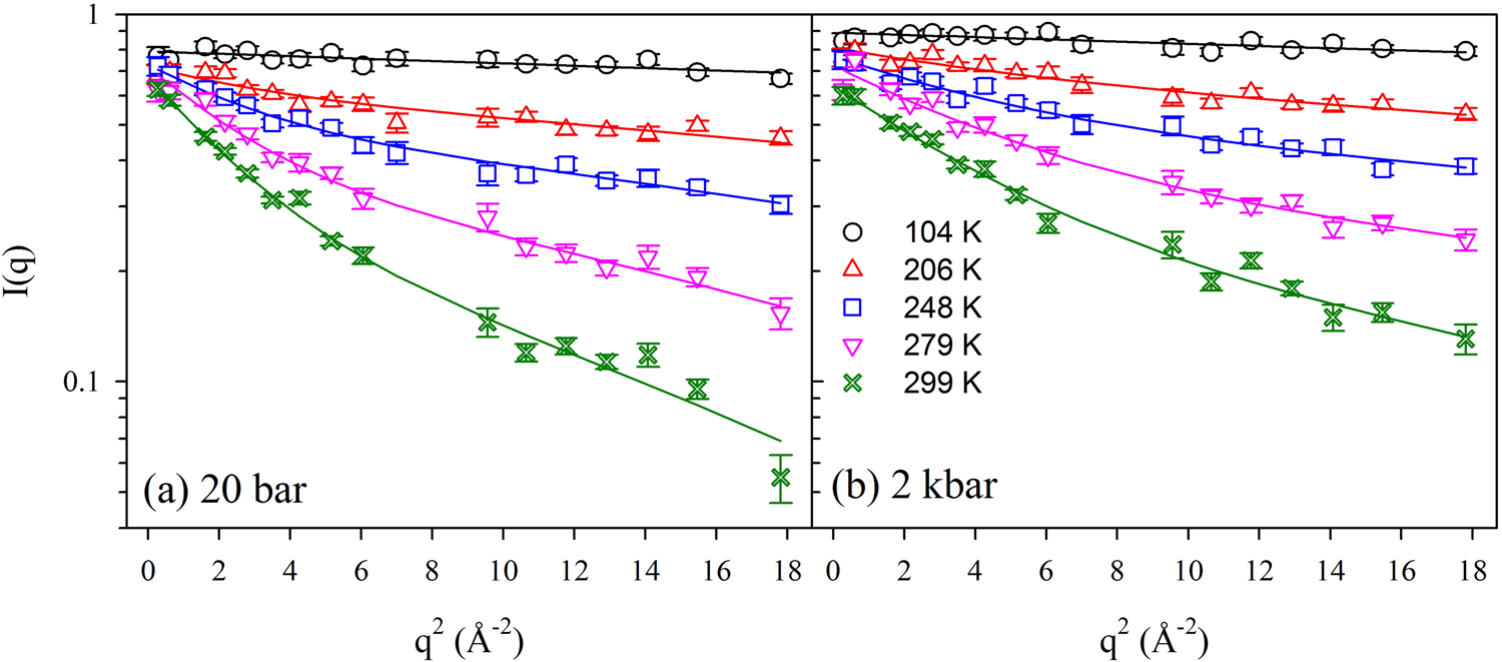
Scattering data and fitting according to eq. 2 for the sample at a pressure of 20 bar (**a**) and of 2 kbar (**b**).

High pressure neutron scattering data are in general more noisy than in standard experiments, because of the absorption and the relatively strong background signal of the pressure cell^21^. Given this fact, we analysed our data by using the smallest possible number of free parameters. Therefore, at each pressure, we performed a global analysis in terms of eq. 2 of all scattering curves obtained at the different temperatures, assuming an Arrhenius behaviour for the populations of the two states (${p}_{2}/{p}_{1}=\exp [-{\rm{\Delta }}G/(RT)]$, where *R* is the gas constant) and a temperature independent distance *d* between the two wells. Excellent fittings were obtained at all temperatures and pressures (examples are shown in Fig. 3). Thermodynamic parameters were found to be largely pressure independent, further confirming that *T*_*on*_ does not depend on pressure. Their values (Δ*H* = (9.7 ± 0.5) kJ/mol and Δ*S*/*R* = 2.5 ± 0.3) are in reasonable agreement with those found by Doster and coworkers in their seminal work on hydrated myoglobin powder^4^.

**Figure 4:**
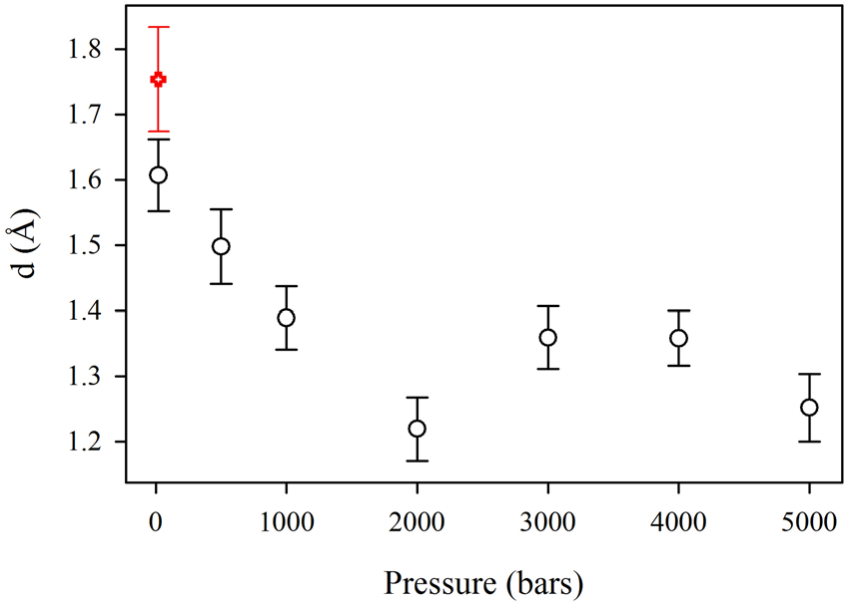
Distance d between the two wells in the two-state model as a function of pressure. The red point refers to the sample at low pressure after the end of the experiments.

The only parameter which is strongly affected by pressure is the distance *d* between the two wells, which is reported as a function of pressure in Fig. 4. At low pressure, we find the same value obtained for hydrated powders^4^. The behaviour with pressure is very similar to the one observed for the total MSD (Fig. 1b), indicating that, within the framework of the two-state model, the reduction of the total MSD brought about by increasing pressure is entirely due to a corresponding reduction in the separation between the two wells. Also the increase in the total MSD following pressure induced denaturation seems to be due to an analogous increase in the distance between the two wells, as shown by the value obtained at low pressure, after the end of the experiments (red point in Fig. 4).

As said before, the two wells in the two-state model can be considered as an average representation of the very large number of different minima in the free energy landscape of the system. In this context, our data indicate that the reduction with pressure of the non-harmonic contribution to molecular motions arises from the fact that at high pressure protein molecules explore CS characterized, on the average, by smaller structural differences between each other. The pressure independence of *T*_*on*_ indicates that high pressure values do not change neither the free energy difference between CS nor the barriers among them. As a consequence, we can infer that the transitions among different statistical substates do not imply significant variations in the volume occupied by the protein molecule, or, at least, that at high pressure values, only those transitions which do not imply significant volume variations are allowed, in agreement with the observed smaller value for the distance between wells in the two-state model. It must be noted that a sizeable effect of pressure on the populations of high tier CS was reported, in the case of the A substates of myoglobin^43^. This clearly implies a corresponding effect of pressure on the free energy differences between these substates and strongly suggests that a change of volume must occur in the transition between them. The reason is that the main difference between A substates of myoglobin resides in the position of a single residue, the distal histidine, in or out from the heme pocket, obviously involving a volume variation. In our case, the MSD obtained by neutron scattering are most likely affected by fluctuations among low tiers –statistical– substates, with completely different properties. In this case, i.e. for low tiers substates, it is perfectly plausible that we have transitions without significant volume variations, as indicated by our data.

**Figure 5:**
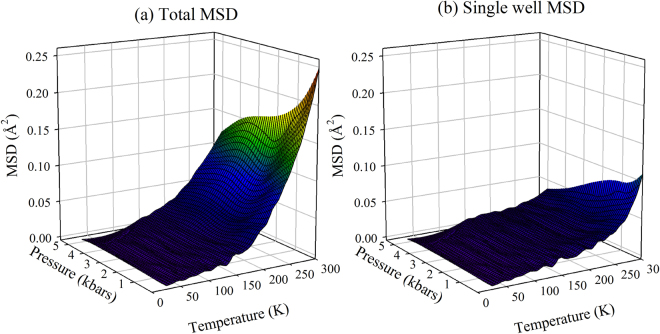
MSD, as obtained from the analysis in terms of the two-state model, as a function of temperature and pressure. (**a**) Total MSD; (**b**) MSD inside each single well.

**Figure 6:**
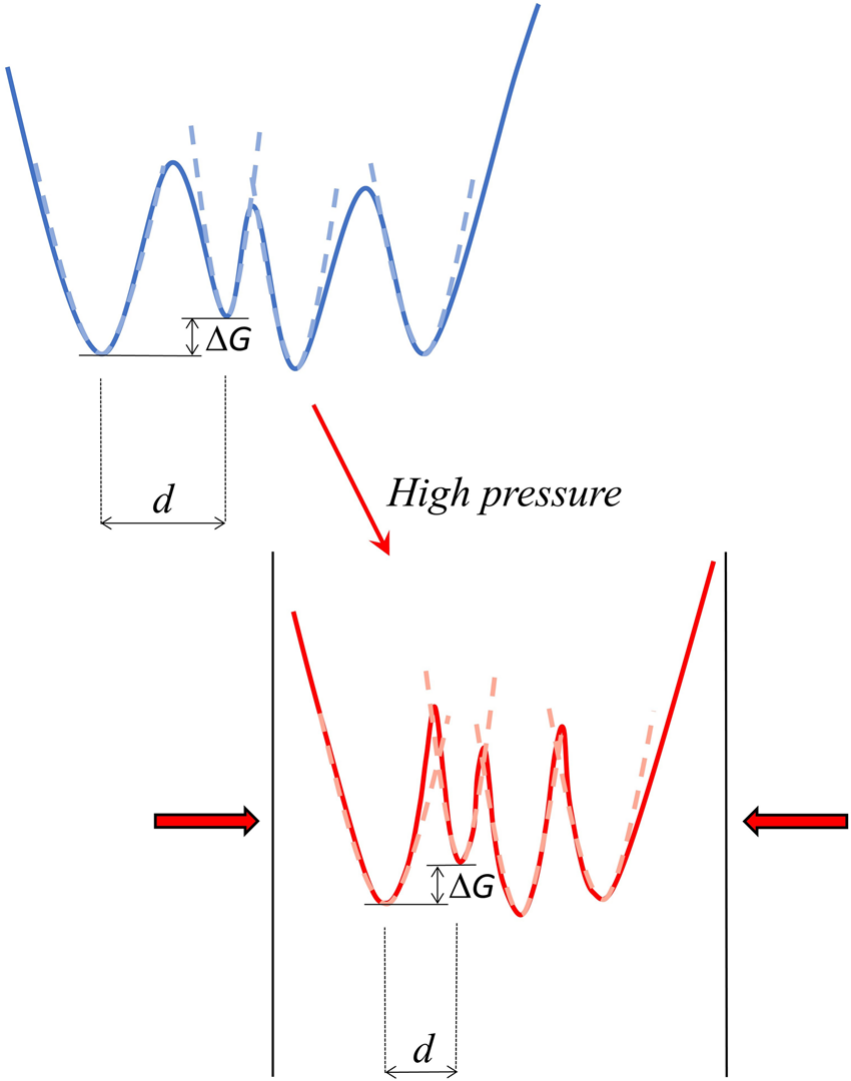
Schematic representation of the effects of pressure on the protein energy landscape.

The analysis in terms of the two-state model enables to estimate the average distance and the average energy difference between “neighbouring” CS in the free energy landscape of the protein; only the average distance is affected by pressure. In Fig. 5 we report the MSD values obtained from the analysis in terms of the two-state model, both total and inside each single well (respectively ${\langle {\rm{\Delta }}{x}^{2}\rangle }_{tot}$ and ${\langle {\rm{\Delta }}{x}^{2}\rangle }_{0}$ in eq. 3). As evident in Fig. 5b, also for the MSD inside the single wells we observe at high temperature some anharmonic contributions (i.e. deviations from the linear behaviour with temperature), which are reduced at high pressure values. Pressure-induced denaturation seems to have on the MSD inside each single well a smaller effect, when compared to what observed for the total MSD. Data in Fig. 5 show that the low temperature harmonic behaviour and *T*_*on*_ do not depend on pressure, both for MSD inside each well and for total MSD. A pressure independent harmonic behaviour indicates that the curvature of the energy landscape close to its minima is not affected by a pressure increase, a result in contrast with the Molecular Dynamics results on hydrated lysozyme^27^. Further studies are needed to clarify this point. In summary, the effects of pressure on the energy landscape, as inferred from the analysis in terms of the two-state model, can be sketched as reported in Fig. 6. High pressure values lead to a reduction in the average structural differences between conformational substates, without any effect on the energy differences and barriers among them, and without effects on the curvature of the landscape close to its minima.

Since the two-state model could be considered as an undue oversimplification of the complex energy landscape of proteins, a further discussion of our results from another perspective is highly desirable. This can be achieved by considering the MSD as arising from a random “diffusion” process occurring, within the time scale of the experiments, in a rugged protein energy landscape^2,44,45^. In this context, Fractional Brownian Diffusion (FBD) has been proposed as a valuable tool to model protein dynamics^46–50^. Within this frame, a sub-diffusive behaviour of the MSD has been found as a function of time, i.e. $\langle {\rm{\Delta }}{x}^{2}(t)\rangle  \sim D{t}^{\alpha }$, where *D* is an effective diffusion coefficient and *α* is the exponent characterizing the diffusion behaviour (we have normal diffusion for *α = *1, and sub-diffusion when *α < *1), and the proportionality coefficient depends on the dimensionality of the motions. In the above expression, *D* is an effective diffusion coefficient that depends on the ratio δ^2^/Δt, where δ is the average length of each random step and Δt is the average time interval between jumps, related to the “friction” experienced by the diffusing particle^51^. A sub-diffusive behaviour may arise from a random walk in a distribution of hierarchically arranged energy barriers^52,53^. In their FBD studies of the effects of pressure on the dynamics of lysozyme, Kneller and co-workers found that high pressure (3 kbar) reduces the extent of MSD to about 70% of their atmospheric pressure values, but maintaining the same identical sub-diffusive behaviour, with an exponent α = 1/2, not dependent on pressure^46–48^. To compare with our data, we note that our Mean Square Displacement can be expressed as MSD ~ *Dτ*^*α*^, where *τ* is the time resolution of the neutron scattering spectrometer used (*τ* ≈ 100 ps for IN13), and that, at P = 3 kbar, we also observe a MSD reduction to about 70% (see Fig. 1b). The above observations strongly suggest that the distribution of barriers bringing about the subdiffusive behaviour of the MSD does not change with pressure, in agreement with the fact that the onset temperature of the PDT is pressure-independent, and that the reduction in the extent of the MSD can be ascribed to a decrease of the average length of each random step or to an increase of the “friction”, or to both effects. This is clearly in agreement with the conclusion that, at high pressure, protein dynamics is determined by a more rugged accessible energy landscape, without substantially altering the barrier heights or their heterogeneity. Therefore, analysis in the framework of a random walk process in a rugged energy landscape corroborates the results of the two-state model analysis, while suggesting a possible role of friction in the pressure effects on protein dynamics.

A rugged energy landscape can clearly have a strong influence on the sub-nanosecond dynamics, as in our case, but it may also affect motions occurring on much longer time scales, up to those required for protein function or even for processes like folding and unfolding. It must be noted, in this respect, that recent works give more and more hints towards the understanding that the dynamics in proteins is coupled over decades in time and self-similar^54,55^. In fact, an increased roughness in the energy landscape has been suggested in studies on the effects of pressure on protein folding, to explain the slowing down of the folding and unfolding kinetics^28–30^, and also for the increased damping of collective motions observed by molecular dynamics simulation^27^. From our data, denaturation also seems to affect the roughness of the energy landscape accessible to proteins, an observation which claims for further investigations, also for its possible link with the presence of cavities in the folded form of the protein^41^.

In conclusion, our data clearly show that, independent on the model and detailed analysis used, high pressure strongly affects protein dynamics only above the PDT; pressure effects consist mainly in a reduction of the observed MSD and can be traced to a decreased average distance between “neighbouring” minima of the protein energy landscape, which means an increased roughness of the landscape and/or increased friction. For these important findings, we have here a strong experimental evidence on a system, myoglobin-glycerol-water, which is a paradigm for studies on the dynamical properties of proteins and complex systems. To further extend the study, it would be of extreme interest to perform the same experiments and analysis on protein systems obtained from organisms adapted to extreme pressure conditions^25,26^.

## Materials and Methods

### Sample preparation

Horse skeletal muscle myoglobin and D_2_O were purchased from Sigma-Aldrich and used without further purification. D_8_-Glycerol was purchased from Cortecnet Europe (Voisins-Le-Bretonneux, France). Myoglobin was firstly dissolved in a D_2_O solution (50 mM phosphate buffer, pD 7.4) to allow H-D exchange, and then lyophilized after two-days equilibration. Following the procedure indicated by Jansson *et al*.^31^, the sample was prepared by successive slow additions of 500 mg of protein in 500 mg of a mixture of D_8_-Glycerol (67%) and D_2_O (33%). During the successive additions, the sample was gently mixed under nitrogen atmosphere, to allow protein solubilization. Since the sample obtained in this way was not completely homogeneous, excess D_2_O (500 mg) was added for a complete protein dissolution, and then removed by lyophilization until the desired sample weight and hydration were reached. At the end of preparation, the sample had the following weight proportions: Myoglobin 50%, D_8_-Glycerol 33.5%, D_2_0 16.5%. This gives a total hydration h = [grams of solvent]/[grams of protein] = 1, and a ratio D_2_O/protein ~0.33. 500 mg of the above sample were used for the measurements.

### High Pressure Neutron Scattering Measurements

Elastic Incoherent Neutron Scattering experiments were performed on the IN13 backscattering spectrometer^56^, at ILL, Grenoble. At the elastic scattering position, IN13 makes use of a 2.23 Å incoming neutron wavelength and 0.2 to 4.9 Å ^1^ momentum transfer, with an energy resolution of 8 μeV, allowing to probe local motions up to ~100 ps. To obtain the intensities scattered by the sample, the scattering from the empty high pressure cell was subtracted. For each pressure value, the data were normalized to the corresponding scattering intensity obtained at the lowest temperature (20 K). Data reduction was carried out using the LAMP software, available at ILL^57^. As a preliminary analysis, the total Mean Square Displacements $\langle {\rm{\Delta }}{x}^{2}\rangle$ of non-exchangeable hydrogen atoms were obtained by using, in the q-range 0–2 Å ^1^, the Gaussian Approximation^33^:

4 $$I(q)\approx {e}^{-{q}^{2}\langle {\rm{\Delta }}{x}^{2}\rangle }$$

Over the past years, a new High Hydrostatic Pressure (HHP) equipment for biological samples in solution was developed in collaboration with the sample environment team SANE of the ILL. It consists of a pressure controller, which communicates with the instrument control software NOMAD, the high pressure stick^58^ and the cell^59^. Pressure is transmitted hydrostatically from the controller to the sample through a capillary using the Fluorinert^TM^ liquid^60^ that has a pour point of 178 K. As the stick is inserted in the cryostat to regulate and control temperature, one has to avoid that liquid freezes at the cold point of the cryostat, therefore it must be heated by a wire and isolated thermally from its environment by a secondary vacuum. The HHP cell is cylindrical, built of the high-tensile aluminium alloy 7075-T6, with an external diameter of 15 mm and an internal diameter of 6 mm. It withstands pressure up to 5 kbar. The sample solution was separated from Fluorinert^TM^ by a separator on the top of the cell. As a sample thickness of 6 mm would lead to multiple scattering and thus to the loss of information about the scattering angle, an Al cylinder of 4 mm diameter was inserted in the middle of the cell.

The same sample was used for all measurements at the different pressure values. The elastic scattering intensity was measured in the temperature range 20–300 K, and for pressure values from 20 bar to 5 kbar. For each pressure, starting from low to high values, the sample was firstly equilibrated for the pressure at 300 K and then brought down to 20 K, while keeping constant the pressure. The scattering intensity was continuously measured when going up in temperature at a rate of 0.26 K/min. Data were successively binned up, by attributing the average temperature value to the scattering curves.

After the measurement at the highest pressure value (5 kbar), the sample was brought again to low pressure (20 bar), and the scattering intensity was measured, only at high temperature, to check for hysteresis, or irreversible phenomena, after exposure to high pressure values.

### Data availability

Data generated during the current study are available from the corresponding author on reasonable request.

## Electronic supplementary material

Supplementary information
