# Supplementary material for: The effects of pressure on the energy landscape of proteins
Source: Sci Rep. 2018 Feb 1;8:2037. doi: 10.1038/s41598-018-20417-x (PMC5794985; doi:10.1038/s41598-018-20417-x)
Supplement: Supplementary file 1 — Supplementary information [file 41598_2018_20417_MOESM1_ESM.pdf]

The effects of pressure on the energy landscape of proteins.

Fabio Librizzi, Rita Carrotta, Judith Peters, Antonio Cupane

Supplementary Information

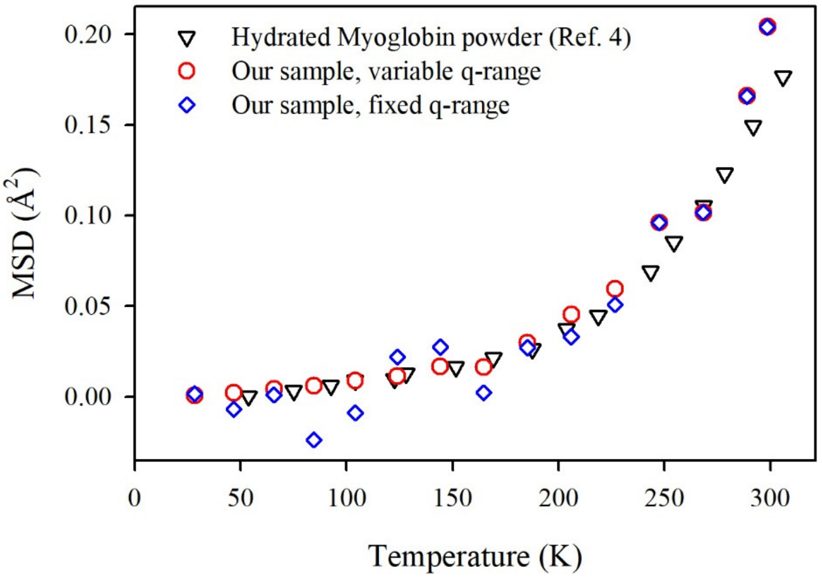

Comparison between the total Mean Square Displacements (MSD) relative to myoglobin in our sample at low pressure and in standard hydrated protein powders (data taken from [1]). For our sample, the total MSD was calculated by applying the Gaussian approximation in two distinct ways: 1) by selecting, for each temperature value, a suitable q- range, so that $(6\langle\Delta x^2\rangle_{tot} \cdot q^2_{max})$<2 (red circles) [2-5]; 2) by considering, for all temperatures, a unique small q-range (0-2 Å^{-1}, blue diamond). The values obtained by using a variable q-range, always satisfying the Gaussian approximation, (red circles) are less affected by noise at low temperature, because the small values of the Mean Square Displacements allow to take into account a larger q-range. The good agreement with the data relative to hydrated myoglobin powders confirms the appropriateness of our experimental conditions.

1. Doster, W., Cusack, S. & Petry, W. Dynamical transition of myoglobin revealed by inelastic neutron scattering. *Nature* **337**, 754-756 (1989).
2. Rahman, A., Singwi, S. & Sjölander, A. Theory of slow neutron scattering by liquids. I. *Phys. Rev*. **126**, 986-996 (1962).
3. Réat, V., Zaccai, G., Ferrand, M. & Pfister, C. in *Biological Macromolecular Dynamics*, 117-122 (Guilderland, NY1997).
4. Réat, V. Patzelt, H., Ferrand, M., Pfister, C., Oesterhelt, D. & Zaccai, G. Dynamic of different functional parts of bacteriothodopsin: H-^{2}H labelling and neutron scattering. *Proc. Natl. Acad. Sci. USA* **95**, 4970-4975 (1998).
5. Zaccai, N.R., Serdyuk, I.N. & Zaccai G. *Methods in molecular biophysics. Structure, dynamics, function for biology and medicine.* (Cambridge University Press, 2017).
